# Aerobic exercise and MOTS-c attenuate diabetic myocardial fibrosis via inhibition of the THBS1/TGF-β signaling pathway

**DOI:** 10.3389/fendo.2026.1732329

**Published:** 2026-02-03

**Authors:** Zhiyu Li, Tutu Wang, Yu Fu, Feilong Chen, Shunchang Li

**Affiliations:** Institute of Sport Medicine and Health, Chengdu Sport University, Chengdu, China

**Keywords:** aerobic exercise, diabetes, diabetic myocardial fibrosis, MOTS-c, transcriptomics

## Abstract

Myocardial fibrosis stands as a defining pathological characteristic in type 2 diabetes. Aerobic exercise curbs the overproduction of type I/III collagen and boosts matrix metalloproteinases, thereby improving outcomes related to fibrosis in the heart. Although physical activity is well known for its positive effects on cardiac well-being in diabetic individuals, sticking to an exercise regimen proves to be a tough nut to crack for many patients. The mitochondrial peptide MOTS-c could serve as a stand-in for exercise, delivering cardioprotective benefits akin to those from working out. This study delved into the molecular mechanisms behind how exercise and MOTS-c influence cardiac fibrosis in diabetes. Both approaches led to marked enhancements in glucose and lipid metabolism, lowered collagen buildup, and bettered both systolic and diastolic heart functions. Through transcriptomic profiling, THBS1 emerged as a pivotal gene that was altered, with reduced activity in the THBS1/TGF-β pathway verified at mRNA and protein stages. In a nutshell, these insights indicate that aerobic exercise and MOTS-c alleviate myocardial fibrosis in diabetes, most likely by putting a damper on the THBS1/TGF-β signaling pathway. These findings establish a theoretical foundation for treating diabetic myocardial fibrosis.

## 1 Introduction

Type 2 Diabetes Mellitus (T2DM) T2DM, a major international health issue, impacts 537 million individuals globally, accounting for roughly 10.5% of the global populace(Zheng, Ley and Hu, 2018). Diabetic cardiomyopathy (DCM) involves a gradual deterioration of cardiac performance, resulting in heart failure (1). Key features of DCM include impaired left ventricular diastolic function, reduced ejection fraction, ventricular hypertrophy, and interstitial fibrosis (2). Early detection of myocardial fibrosis and timely intervention in fibrotic pathways are crucial for effectively managing DCM, given the pivotal role of fibrosis in disease progression (3).

Physical activity enhances insulin sensitivity, reduces blood glucose and cholesterol levels, and is crucial for the prevention and management of DCM. Exercise also exerts a positive impact on cardiac structure and function in diabetic individuals, making it a key component of clinical strategies for managing DCM (4). Endurance training in DCM patients can alleviate left ventricular remodeling, improve both systolic and diastolic cardiac functions, and enhance mitochondrial function, antioxidant capacity, and myocardial cell metabolism (5).

Discovered in 2015 by Lee, MOTS-c is a 16-amino acid peptide derived from mitochondrial 12S rRNA gene coding (6). This peptide is released from mitochondria and circulates through the bloodstream to various organs, exerting systemic effects (7). Predominantly found in the myocardium, skeletal muscle, serum, and brain, MOTS-c serves as a vital cellular protector. Interestingly, when you’re breaking a sweat, this molecule steps up to the plate, ensuring that metabolic balance is kept in check, shielding mitochondria from harm, and supporting overall cell well-being (8–10). Whether through physical exercise or supplementation, MOTS-c can trigger beneficial adaptive responses in the organism. Previous research confirms that MOTS-c alleviates diabetic myocardial damage via antioxidant, anti-inflammatory, and oxidative stress reduction mechanisms (11–14). These results demonstrate MOTS-c’s remarkable ability to replicate exercise-induced benefits, revealing its crucial function in regulating the body’s physiological reactions to physical activity. The peptide essentially acts as an exercise mimetic, offering similar advantages without requiring actual physical exertion. This discovery positions MOTS-c as a key player in metabolic regulation and cellular adaptation processes typically triggered by workout routines.

Our earlier study showed that MOTS-c replicates the positive impacts of exercise on diabetic rats’ heart function, yet the specific molecular route is still enigmatic. Therefore, we turned to transcriptomics to shed light on the specific processes through which MOTS-c alleviates heart muscle scarring in diabetic conditions. The study aimed to assess the effects of different eight-week treatments on diabetes-induced cardiac fibrosis. By employing transcriptomic profiling, we sought to unravel the intricate mechanisms at play and pinpoint promising therapeutic avenues for diabetic cardiomyopathy.

## 2 Materials and methods

### 2.1 Experimental animals

Sixty 8-week-old Sprague-Dawley rats, weighing in at 180 to 200 grams, were sourced from the reputable Chengdu Dashuo Experimental Animal Technology Co., Ltd. in Chengdu, China, and they were confirmed to be SPF-grade. The treatment and experimentation protocols were ethically reviewed and given the green light by the Experimental Animal Ethics Committee at Chengdu Sport University (Approval Number: [2021]7).

Each rat had its own cozy home within a cage that typically houses four, thanks to the accommodations at Chengdu Sport University’s animal facility. They had unrestricted access to nourishment and hydration. The facility maintained the highest standards of animal care, with temperatures kept at a comfortable 22 ± 2°C, a humidity level of 40-60%, and a schedule that mimics the sun’s daily rise and fall.

### 2.2 Experimental grouping

**Figure 1:**
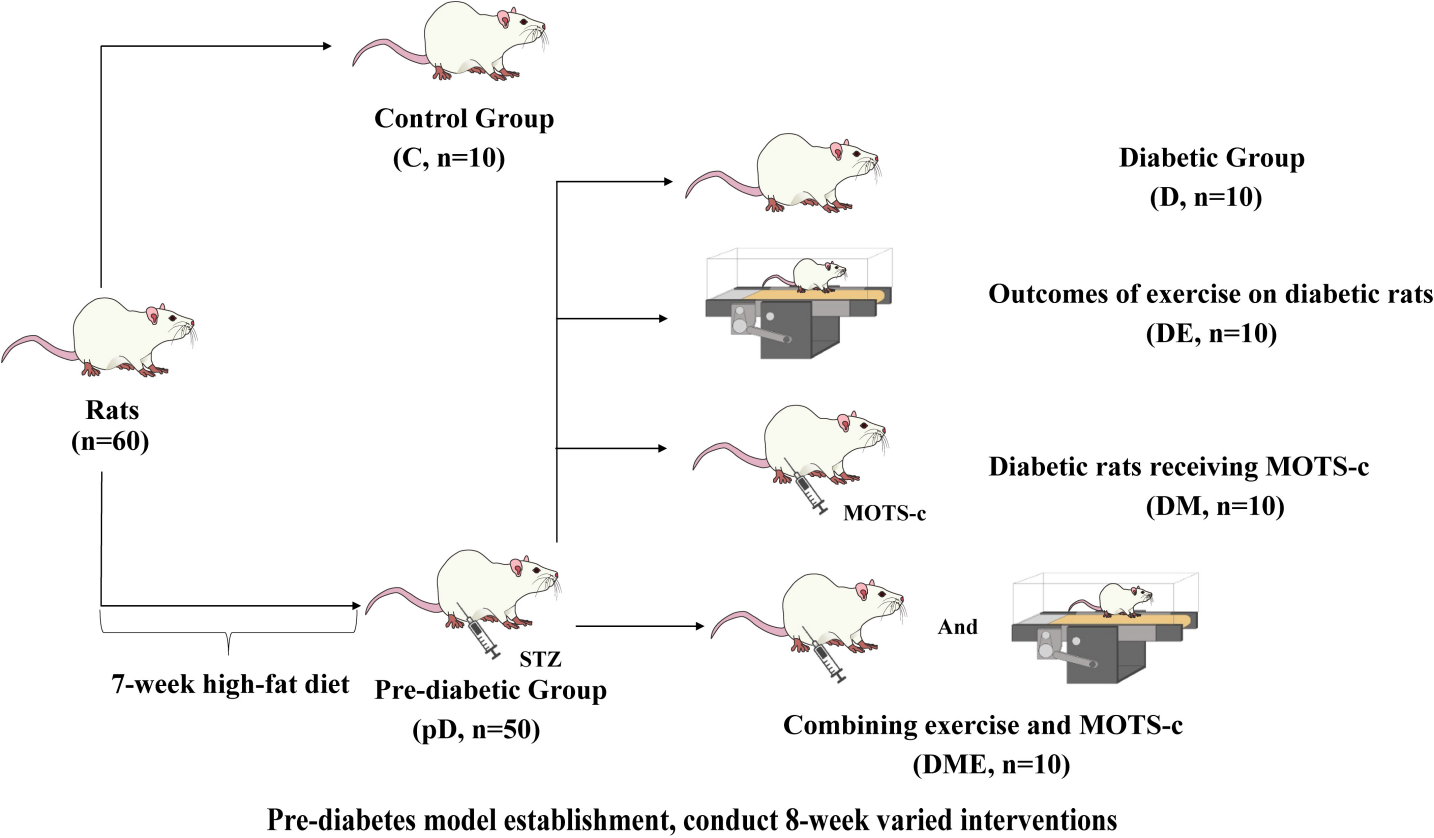
Experimental grouping. Rats were randomly divided into 5 groups: (1) control group (C, n = 10), (2) diabetes group (D, n = 10), (3) outcomes of exercise on diabetic rats (DE, n = 10), (4) diabetic rats receiving MOTS-c (DM, n = 10), (5) combining exercise and MOTS-c (DME, n = 10).

The researchers divided the rats into two distinct cohorts: a control group comprising 10 subjects and a pre-diabetic group of 50 animals. Following a seven-week high-fat diet regimen, the pre-diabetic subjects received an intraperitoneal streptozotocin injection (30 mg/kg) dissolved in sodium citrate buffer, while the control group was administered an equivalent volume (0.25 mL/kg) of the buffer solution alone. To confirm the induction of diabetes, blood glucose measurements were taken 72 hours post-injection, with hyperglycemia formally established when glucose levels surpassed 16.7 mmol/L (15). After diabetes induction, 50 pre-diabetic rats were randomly distributed across 4 new groups. Baseline characteristics of experimental mice showed no significant differences in group testing (**Table 1**). The control group (C, n = 10) served as the baseline, while the diabetes group (D, n = 10) suffered from the condition. Additionally, we examined the outcomes of exercise on diabetic rats (DE, n = 10) and diabetic rats receiving MOTS-c (DM, n = 10). To delve deeper, we also analyzed the impact of combining exercise and MOTS-c (DME, n = 10) (**Figure 1**).

**Table 1:** Baseline characteristics of rats before grouping.

Group	FBG (mmol/L)	FINS (mU/L)	HOMA-IR
control	6.82 ± 0.65	14.52 ± 2.25	2.31 ± 0.03
pre-diabetic	8.21 ± 0.45^**^	10.23 ± 1.82^**^	3.89 ± 0.04^**^

^*^p < 0.05, ^**^p < 0.01, compared with group control.

### 2.3 Exercise intervention protocol

Rats in the groups DE and DME adhered to the prescribed exercise regimen based on the Bedford (16) exercise model on a treadmill. Before the study’s start, the rodents had to get used to the setup over a three-day period. Each day, they spent 15 minutes on the task, cruising at about 10 to 15 meters per minute. Once the actual training began, the rats hit the tracks for an hour each day, five days a week, for a solid eight weeks. They kept up the pace at 15 meters per minute. To keep things steady, the researchers used some high-tech driving aids and tail tickling to make sure the speed was just right.

### 2.4 MOTS-c injection intervention

The DM and DME groups of rats were administered MOTS-c via intraperitoneal injection at a daily dose of 0.5 mg/kg (8, 12, 13, 17), delivered five times per week over an eight-week period. In contrast, the control groups received equivalent volumes of normal saline through the same injection method and schedule. The MOTS-c compound was custom-synthesized through *in vitro* methods by GL Biochem (Shanghai) Ltd., located in Chengdu, China.

### 2.5 Evaluation of blood glucose, insulin levels, and insulin resistance

Blood sugar levels were quantified using a standard glucose meter produced by ACON Biotech in Hangzhou, China. For serum insulin measurements, scientists employed a rat-specific ELISA kit sourced from ImmunoWay Biotechnology in San Jose, California. A Thermo Fisher Scientific SpectraMax M5 plate reader (Waltham, MA) was used to capture absorbance values at 450 nm. To assess insulin resistance, the team calculated HOMA-IR scores through the standard equation: (Fasting Glucose × Fasting Insulin)/22.5, following established protocols for evaluating metabolic dysfunction (18).

### 2.6 Echocardiography

Echocardiograms were performed using an animal echocardiograph (Philips CX50, Holland) following an 8-week intervention. The study evaluated several key cardiac measurements, such as LVIDd and LVIDs, along with LVPWd. Additional parameters included EF, FS, E/A, and LVPWs. These metrics provided a comprehensive assessment of heart function.

### 2.7 Sample collection

To minimize the acute effects of the exercise intervention, experimental samples were collected 48 hours after the final training. The rats were anesthetized via intraperitoneal injection of 3% pentobarbital sodium at a dosage of 0.15 ml per 100 grams of body weight. While completely under, blood samples were collected directly from the abdominal aorta, with serum subsequently obtained. Cardiac tissue was promptly extracted, meticulously cleansed, and preserved for further examination. Select tissue specimens were fixed for histological evaluation using both hematoxylin and eosin (HE) staining and Masson staining protocols. The remainder of the cardiac tissue was secured in tin foil-lined cryotubes and stored at -80°C to be utilized in subsequent investigations.

### 2.8 Blood lipid analysis

Blood was drawn into ethylene propylene (EP) tubes and subjected to centrifugation at a rate of 3000 revolutions per minute (rpm) for a duration of 10 minutes, allowing the separation of the supernatant. This process was conducted with the aid of a centrifuge model CHT210R, which originated from Hunan, China. Subsequently, TG, HDL-C, LDL-C, and TC levels were quantified via an automated biochemical analyzer known as the Mindray BS-280M, which hails from Shenzhen, China.

### 2.9 HE staining

The tissue specimens first underwent deparaffinization and rehydration before being subjected to HE staining. The nuclei were initially treated with Harris’s hematoxylin, followed by differentiation in acid alcohol and subsequent bluing with ammonia water. For cytoplasmic staining, we applied eosin Y solution for a period of 1–3 minutes. Samples underwent ethanol dehydration, xylene clearing, and neutral balsam mounting prior to microscopic analysis.

### 2.10 Masson staining

After preparing thin tissue slices preserved in paraffin, we employed Masson staining to visualize tissue components. This protocol began with removing the paraffin and gradually rehydrating the samples before treating them with Bouin’s solution at 56°C for one hour to ensure proper fixation. To highlight cellular structures, we first applied Weigert’s iron hematoxylin to stain the nuclei, followed by Biebrich scarlet-acid fuchsin to color the cytoplasm. For collagen detection, we utilized a solution of phosphomolybdic and phosphotungstic acid to differentiate connective tissue, which was then counterstained with aniline blue. The finishing touches involved a quick rinse in 1% acetic acid, a series of dehydration steps, clarification in xylene, and finally mounting the specimens with synthetic resin to prepare them for microscopic examination.

### 2.11 Transcriptome and functional enrichment analysis

Transcriptome sequencing was performed on mRNA extracted from tissues. A cDNA library was prepared through reverse transcription and sequenced using the Illumina high-throughput platform. RNA extracted from tissues underwent transcriptome sequencing via Illumina platform after cDNA library preparation through reverse transcription. Then, five transcriptome libraries were constructed. We carried out differential gene expression analysis by applying a threshold of corrected p-values below 0.05 in conjunction with an absolute log_2_ fold change of at least 1.2. To further investigate the functional implications, we use clusterProfiler software to conduct both Gene Ontology (GO) and Kyoto Encyclopedia of Genes and Genomes (KEGG) enrichment analyses.

### 2.12 RNA isolation and quantitative real-time PCR

To isolate total RNA from the tissue samples, we employed the DP419 RNA extraction kit from Tiangen Biotech in Beijing, China. This was followed by first-strand cDNA synthesis with the RevertAid kit from Thermo Fisher Scientific. The subsequent RT-qPCR was conducted with custom primers listed in **Table 2**, using the CFX96 system and the GoTaq real-time PCR system. Gene expression levels were determined using the Bio-Rad CFX Manager software and the ΔΔCt method (**Table 2**).

**Table 2:** Primer sequences.

Primer sequences		
Gene	Forward (5’- 3’)	Reverse (3’- 5’)
THBS1	AAGACGTCGACGAGTGCAAA	CCTGTTTGTTGGCCATAGCG
β-Actin	AGCCATGTACGTAGCCATCC	GACTCCATCACAATGCCAGT

### 2.13 Western blotting

We extracted proteins from left ventricular samples and separated them via SDS-PAGE, subsequently transferring the resolved proteins onto PVDF membranes. To sidestep any non-specific binding, we treated these membranes with a 5% non-fat dry milk solution for 90 minutes at room temperature. After this blocking procedure, the membranes underwent an overnight incubation at 4°C with primary antibodies designed to detect MOTS-c, THBS1, TGF-β, and β-Actin. Then, we analyzed with ImageJ software. Primary antibodies for THBS1, TGF-β, and β-Actin were obtained from HuaBio Co. (Hangzhou, China), and MOTS-c antibodies were purchased from BioVision Inc. (California, USA).

### 2.14 Statistical analysis

The data analysis was conducted with SPSS (Version 25) and GraphPad Prism 9, following established academic protocols. Results were summarized using means accompanied by standard deviations (SD) as the main descriptive measures. For group comparisons, normally distributed data underwent one-way ANOVA, while non-parametric datasets were assessed via the Kruskal-Wallis test. Statistical significance was determined at p < 0.05, with results below 0.01 considered highly significant.

## 3 Result

### 3.1 Basic data of body weight

Compared to group C, rats in group D exhibited an 18% reduction in body weight (p < 0.01). Additionally, the heart weight index increased by approximately 24% in group D (p < 0.01). Rats in groups DE and DME showed a significant increase in body weight relative to group D (p < 0.01). Furthermore, rats in the DE, DM, and DME groups exhibited a significant reduction in heart weight compared to group D (p < 0.05), alongside a marked decrease in the heart weight index in groups DE and DME (p < 0.01) (**Table 3**).

**Table 3:** Basic data in T2DM rats.

Group	Body weight (g)	Heart weight (mg)	Heart weight index (mg/g)
C	558.00 ± 13.89	1478.00 ± 16.63	2.61 ± 0.06
D	458.67 ± 13.05^**^	1491.00 ± 35.51	3.25 ± 0.04^**^
DE	513.00 ± 14.73^**##^	1401.67 ± 8.62^#^	2.73 ± 0.09^##^
DM	432.75 ± 7.89^**^	1393.67 ± 54.37^#^	3.23 ± 0.13^**^
DME	510.67 ± 10.07^**##^	1389.00 ± 53.45^*#^	2.72 ± 0.08^##^

^*^p < 0.05, ^**^p < 0.01, compared with group C; ^#^*p* < 0.05, ^##^*p* < 0.01, compared with group D.

### 3.2 Glycolipid metabolism

**Figure 2:**
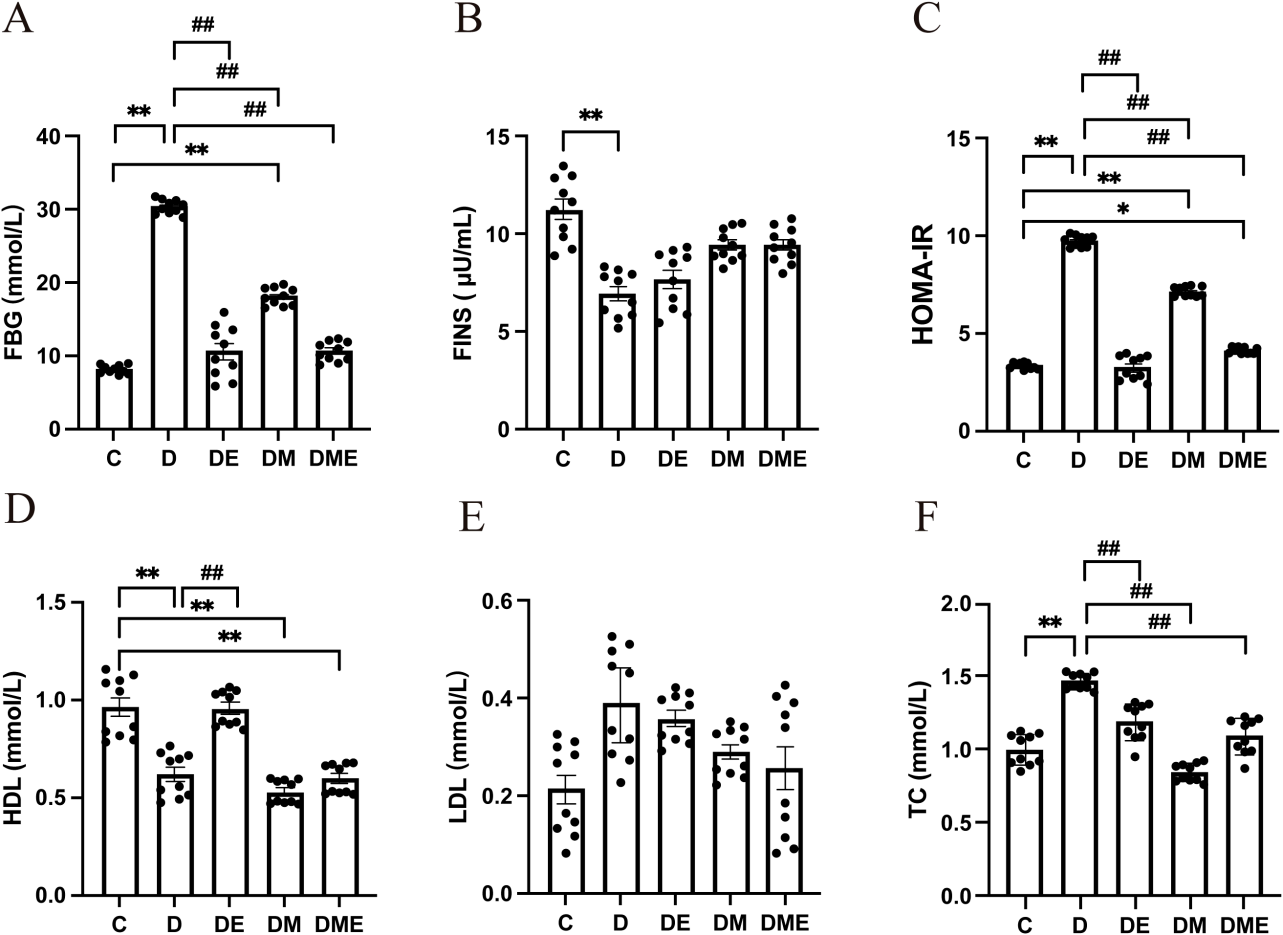
Glycolipid metabolism levels in T2DM rats after an 8-week intervention. **(A)** The FBG level of rats in each group.; **(B)** The FINS level of rats in each group.; **(C)** The HOMA-IR level of rats in each group. Rats from group D had increased HOMA-IR level (p < 0.01), whereas exercise and MOTS-c reduced this ratio (p < 0.01).; **(D)** The HDL level of rats in each group.; **(E)** The LDL level of rats in each group.; **(F)** The TC level of rats in each group. Rats from group D had in-creased TC level compared to rats from group C (p < 0.01), whereas exercise and MOTS-c reduced this ratio in diabetic rats (p < 0.01). *p<0.05, **p<0.01 vs. group C; ^#^p<0.05, ^##^p<0.01 vs. group D.

Rats in group D showed significantly higher Fasting Blood Glucose (FBG), Model Assessment of Insulin Resistance (HOMA-IR), and total cholesterol (TC) levels compared to group C (p < 0.01), with significantly lower levels of high-density lipoprotein cholesterol (HDL-C) and Fasting Insulin (FINS) (p < 0.05). Compared to the D group, the DE group rats showed a marked decrease in fasting blood glucose, HOMA-IR, and TC levels (p < 0.01), accompanied by a significant boost in HDL-C concentrations (p < 0.01). Meanwhile, the DM group demonstrated a considerable reduction in HOMA-IR and TC (p < 0.01), whereas the DME group rats experienced a substantial decline in FBG, HOMA-IR, and TC levels (p < 0.01) (**Figure 2**).

### 3.3 Cardiac function and morphological structure

**Figure 3:**
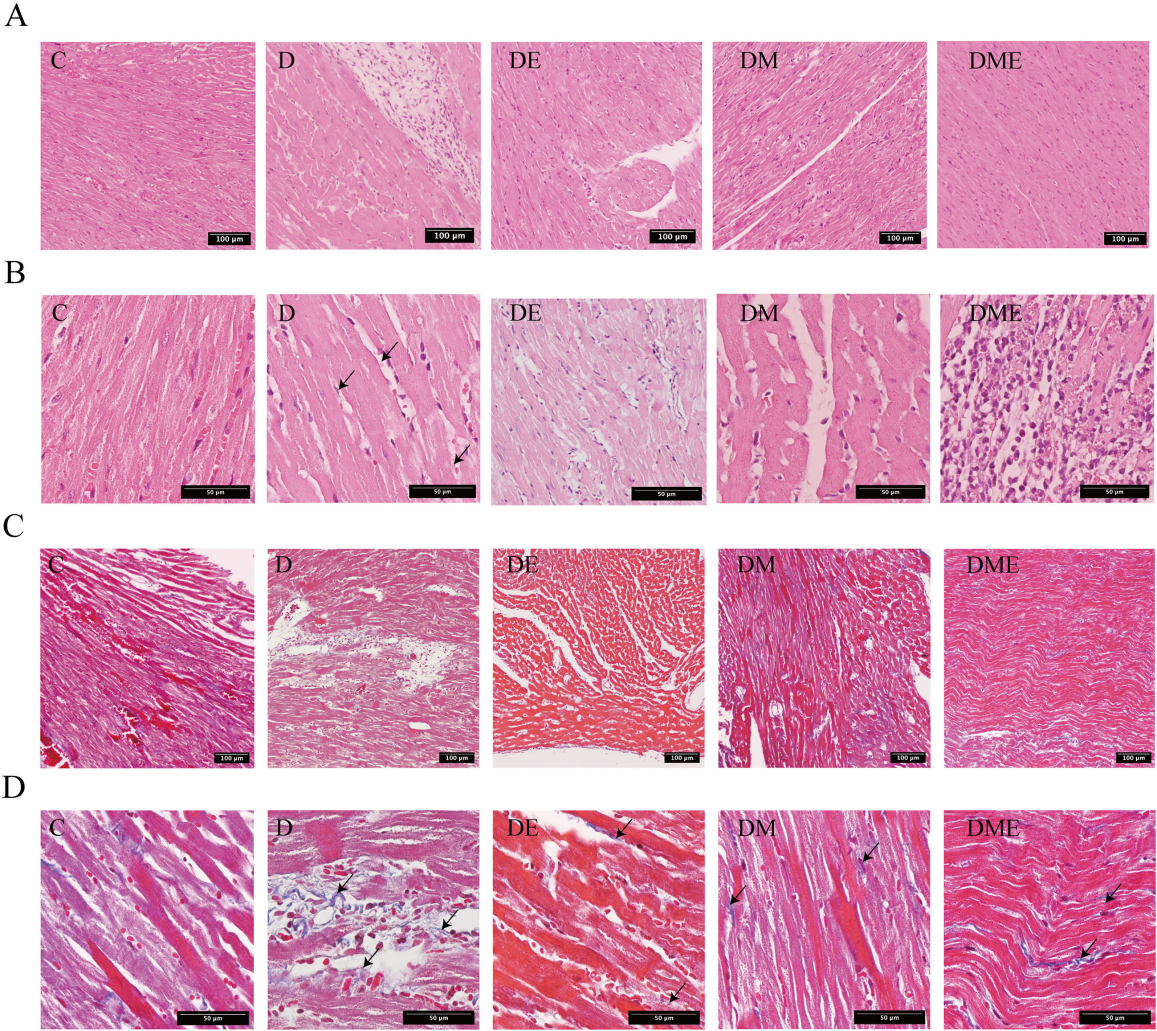
Cardiac morphological and structural alterations in T2DM rats after an 8-week intervention. **(A)** HE staining image of rats in each group (100x).; **(B)** HE staining image of rats in each group (400x). Rats in group D exhibited disorganized myocardial fibers, hypertrophic cells, increased intercellular spaces, indistinct structures, and infiltration of inflammatory cells.; **(C)** Masson staining images of rats in each group (100x).; **(D)** Masson staining images of rats in each group (400x). rats in group D showed significant collagen accumulation.

HE staining results showed that rats in group C had well-organized myocardial fibers with uniform chromatin distribution in cell nuclei, minimal extracellular space, normal microvascular structure, few fibroblasts, and no inflammatory cell infiltration. In contrast, rats in group D exhibited disorganized myocardial fibers, hypertrophic cells, increased intercellular spaces, indistinct structures, and infiltration of inflammatory cells. After exercise and MOTS-c, rats in groups DE, DM, and DME displayed more organized myocardial fibers with no apparent inflammatory cell infiltration (**Figures 3A, B**).

Masson staining indicated no detectable collagen fiber presence in the cardiac tissue of control group C. However, group D demonstrated marked collagen buildup, signaling fibrotic changes. After implementing exercise regimens and MOTS-c treatment, the DE, DM, and DME groups all showed substantial decreases in myocardial collagen deposition (**Figures 3C, D**). Quantitative analysis revealed group D had a strikingly higher collagen volume fraction (CVF) (p < 0.01) compared to controls, confirming fibrosis development. In contrast, the intervention groups (DE, DM, DME) displayed significantly reduced CVF values (p < 0.01) relative to untreated group D (**Table 4**).

**Table 4:** Fibrosis severity in diabetic rats among group comparisons.

Group	CVF
C	32.15 ± 0.82
D	62.05 ± 1.14^**^
DE	37.64 ± 1.38**^##^
DM	50.13 ± 1.00**^##^
DME	45.47 ± 0.12**^##^

^*^p < 0.05, ^**^p < 0.01, compared with group C; ^#^*p* < 0.05, ^##^*p* < 0.01, compared with group D.

**Figure 4:**
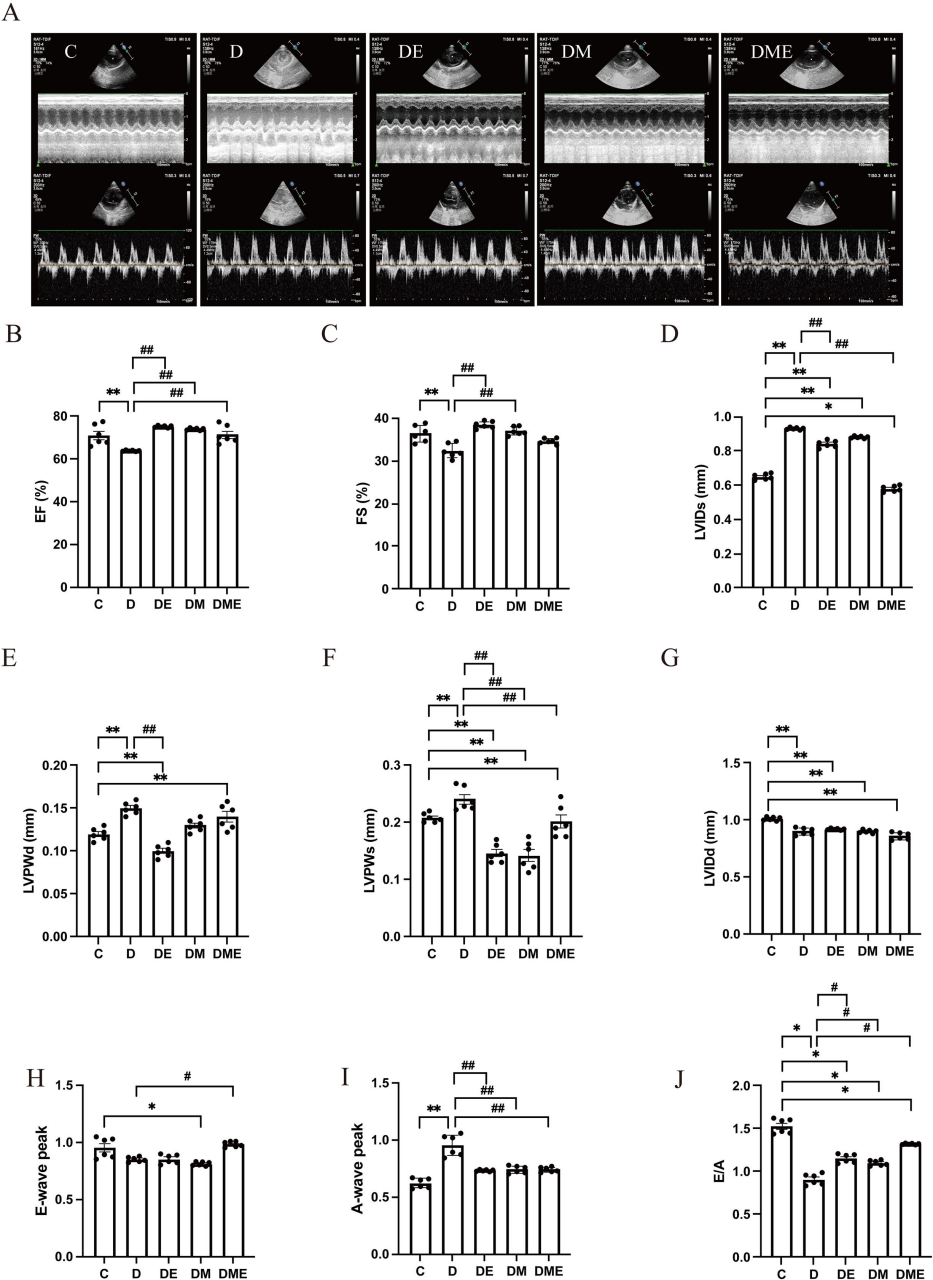
Echocardiographic changes in T2DM rats after an 8-week intervention. **(A)** Echocardiographic images of rats; **(B–J)** Analysis of echocardiographic parameters, including EF, FS, LVIDs, LVPWd, LVPWs, LVIDd, E-wave, A-wave and E/A peak. After 3 interventions, systolic and diastolic cardiac function in rats were improved. ^*^p<0.05, ^**^p<0.01 vs. group C; ^#^p<0.05, ^##^p<0.01 vs. group D.

**Figure 4A** displays characteristic M-mode echocardiographic tracings from experimental groups. Our analysis revealed marked impairments in left ventricular systolic performance (**Figures 4B–I**). Group D animals exhibited statistically significant reductions in both EF and FS (p < 0.05) relative to controls, along with notable increases in LVIDs, left ventricular posterior wall during diastole (LVPWd), and posterior wall thickness during systole (p < 0.05). In terms of diastolic function, Groups D, DE, DM, and DME all demonstrated significantly left ventricular end-diastolic diameter (LVIDd) during diastole and E/A ratios when compared to Group C (p < 0.05). However, the DE, DM, and DME groups showed a partial recovery, with E/A significantly exceeding those of Group D (p < 0.05) (**Figures 4B–J**).

### 3.4 Transcriptome profiling and functional annotation

**Figure 5:**
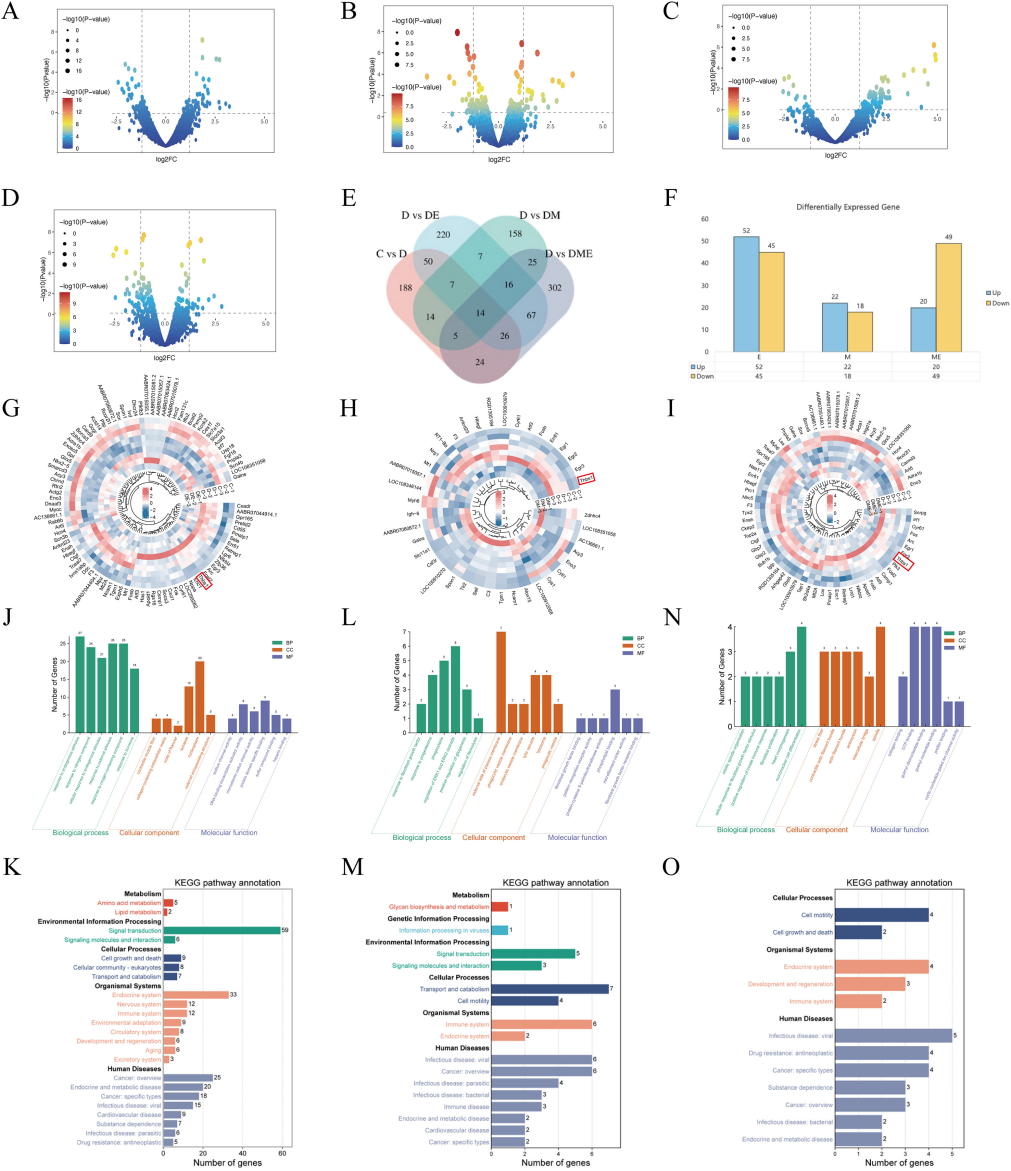
analysis and functional enrichment in diabetes. **(A)** Volcano plot of gene expression variations in diabetes. **(B–D)** Gene Expression Variations Depicted in Volcano Plots: Aerobic Exercise, MOTS-c Administration, and Combined Protocols. **(E)** Venn diagram of gene expression variations among groups. **(F)** Statistical representation of differential gene expression. **(G–I)** Heat maps of gene expression in different groups. **(J–O)** GO and KEGG analysis for the various interventions.

Transcriptomic analysis revealed that diabetes significantly altered the expression of 328 genes, with 167 genes upregulated and 161 downregulated (**Figure 5A**). Aerobic exercise in diabetic rats induced differential expression of 409 genes, with 188 genes upregulated and 221 downregulated (**Figure 5B**). MOTS-c intervention in diabetic rats resulted in differential expression of 246 genes, with 196 genes upregulated and 50 genes downregulated (**Figure 5C**). The combined exercise and MOTS-c intervention led to changes in 480 differentially expressed genes, with 198 genes upregulated and 282 downregulated (**Figure 5D**).

Venn diagram analysis revealed that 97 genes were altered in the group DE, 40 in the group DM, and 69 in the group DME (**Figures 5E, F**). Heat map cluster analysis showed that gene expression patterns in group C were inversely correlated with those in group D, while groups DE, DM, and DME exhibited similar patterns of upregulation and downregulation (**Figures 5G–I**). GO and KEGG analyses revealed that genes in the group DE were enriched in metabolic processes and the extracellular matrix, particularly collagen (**Figures 5J, K**), while genes in the group DM showed enrichment in fibroblast growth factor binding and receptor interactions (**Figures 5L, M**). DME group genes showed heightened activity in fibroblast growth factor response, fibroblast development, and cardiac formation (**Figures 5N, O**).

### 3.5 Effects of aerobic exercise and MOTS-c on THBS1/TGF-β signaling pathway

#### 3.5.1 Detection of THBS1 expression levels

**Figure 6:**
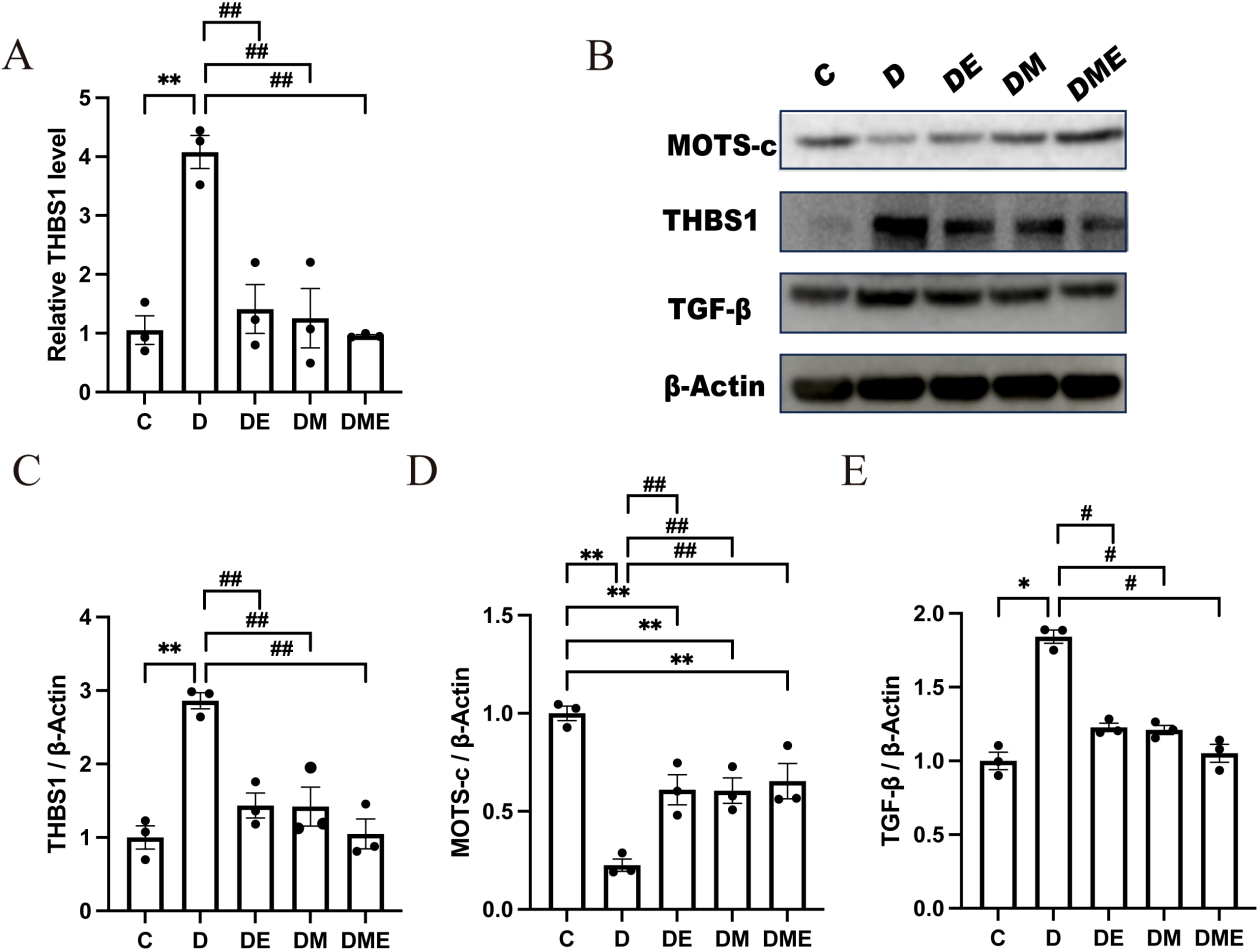
Effects of aerobic exercise and MOTS-c on the THBS1/TGF-β Signaling Pathway. **(A)** The level of THBS1 mRNA expression in each group. **(B–E)** WB analysis and the levels of MOTS-c, THBS1, and TGF-β protein expression. ^*^p<0.05, ^**^p<0.01 vs. group C; ^#^p<0.05, ^##^p<0.01 vs. group D.

Among the genes identified through functional enrichment, THBS1 emerged as a key gene. Our analysis revealed that in diabetic conditions, THBS1 exhibited statistically significant alterations in both P-values and Log2FC, with a notable upward trend. Similarly, following three distinct interventions in diabetic rats, THBS1 demonstrated significant changes in P-values and Log2FC, consistently showing downward trends across all interventions. The implicated pathways encompassed fibroblast growth factor response, fibroblast development, cardiac formation, fibroblast growth factor binding, and receptor interactions. All three interventions implicated THBS1 in mitigating myocardial fibrosis. Considering THBS1 pivotal role in the key enrichment pathways and its consistent, statistically significant reduction in all three intervention groups, we have decided to make it the central target for our subsequent mechanistic validation studies. qRT-PCR analysis confirmed a significant increase in THBS1 mRNA in group D (p < 0.01), with a marked reduction in the groups DE, DM, and DME (p < 0.01) (**Figure 6A**).

#### 3.5.2 Detection of THBS1, MOTS-c, and TGF-β protein expression

The WB analysis validated the protein concentrations for MOTS-c, THBS1, and TGF-β. In group D, there was a notable dip in MOTS-c protein levels when juxtaposed with group C, reaching statistical significance (p < 0.05). Post-aerobic workout, a significant bump was observed in MOTS-c protein levels, especially with the combined intervention, which was also statistically significant (p < 0.05). On the flip side, group D exhibited a considerable surge in THBS1 and TGF-β protein levels (p < 0.05), along with a substantial decline in DE, DM, and DME (p < 0.05), as depicted in **Figures 6B–E**.

## 4 Discussion

### 4.1 Comparative benefits of MOTS-c and cardiovascular activity on diabetic heart fibrosis

In individuals with type 2 diabetes mellitus, persistently elevated blood sugar levels pave the way for heart muscle scarring by spurring cardiac fibroblast growth and amping up the production of proteins that make up the structural framework of heart tissue, which in turn compromises the heart’s ability to function properly (19). Physical exercise reduces the risk of DCM by improving glucose and lipid metabolism and preserving myocardial cellular structure (20). Thus, physical activity significantly impacts both diabetic cardiac disorder prevention and treatment.

Exercise therapy is essential for managing diabetes and its complications; however, adherence to exercise regimens remains a significant challenge. Studies show varying compliance rates among individuals with heart disease, ranging from 40% to 91% (21). Despite 80% of patients acknowledging the benefits of exercise, only 39% adhere to prescribed regimens (22). Research on exercise therapy for heart disease reveals a gradual decline in patient compliance, with adherence rates falling to 32% at 6 months, 28% at 12 months, and 19% at 18 months (23). “Exercise mimetics” now offers a viable means to achieve or surpass exercise advantages for those with inconsistent activity regimens (24). This innovative approach shows potential as a complementary or alternative intervention for such patients.

The term “Exercise mimetics” was first proposed by Himmis-Hagen in 2004 (25), and MOTS-c was discovered by Lee in 2015 (6). Previous studies have demonstrated that physiological stressors, such as exercise, can significantly stimulate the synthesis of MOTS-c, leading to broad health improvements (26). And our previous research has confirmed MOTS-c’s ability to enhance myocardial contractility and cardiac function (11, 17). It’s worth noting that MOTS-c, as a mitochondrial signal peptide present throughout the body, plays a crucial role in understanding its endocrine effects. Existing research has shown that both acute and prolonged aerobic exercise can significantly boost the concentration of MOTS-c in the circulatory systems of both healthy individuals and those suffering from metabolic disorders, such as diabetes(Reynolds et al., 2021; 9, 10). Thus, we hypothesize that modulating MOTS-c could replicate the benefits of exercise, offering a potential strategy to mitigate diabetic cardiac fibrosis.

In line with this, diabetic rats exhibited disorganized myocardial fibers with collagen fiber deposition and elevated CVF. After interventions with MOTS-c, aerobic exercise, and combined treatment, myocardial fibers were more organized, and collagen fiber accumulation was significantly reduced. Additionally, our results show that diabetic rats exhibited significant weight loss, accompanied by marked increases in heart weight index, FBG, HOMA-IR, and TC levels. After the interventions, FBG, HOMA-IR, and TC levels all demonstrated a similar downward trend. MOTS-c exhibited improvements in glucose and lipid metabolism, as well as cardiac structure and function, comparable to aerobic exercise. Extensive research indicates that MOTS-c and aerobic exercise can enhance diabetes and heart conditions by activating the Nuclear Factor Kappa-B (NF-κB)(Liu et al., 2023), AMP-activated protein kinase (AMPK) (6; Xinqiang et al., 2020; Rezaei et al., 2023), and Protein Kinase B (AKT) (Rezaei et al., 2023; Wu et al., 2023)pathways. Our transcriptomic analysis revealed that in the context of diabetes, eight weeks of aerobic exercise and MOTS-c injection interventions, as well as their combined approach, modulated 97, 40, and 69 disease-causing genes, respectively. The comparison between aerobic exercise and MOTS-c injection interventions alone identified 16 overlapping genes, while all three intervention methods shared 11 common genes. Moreover, functional enrichment analysis highlighted the comparable effects of MOTS-c and physical exercise on diabetic myocardial fibrosis. Nevertheless, the joint treatment failed to reveal substantial variation in outcomes against the solitary therapies, likely due to the comparable pathways triggered by MOTS-c and aerobic activity.

### 4.2 MOTS-c and aerobic activity enhance mitigation of diabetic myocardial fibrosis through the THBS1/TGF-β pathway

According to our findings, an eight-week program of aerobic exercise, MOTS-c, or a combination of both interventions significantly enhances glycolipid metabolism in diabetic rats. The data demonstrates that these approaches yield measurable improvements in metabolic function. This intervention not only fortifies the myocardial structure and function but also reduces fibrosis in the heart tissue. Using transcriptomic analysis, we identified key genes modulated by these interventions, providing insights into the underlying molecular mechanisms. Functional enrichment analysis revealed that genes affected by 8 weeks of aerobic exercise are associated with hormone responses, collagen-containing extracellular matrix, and heparin binding. Genes influenced by 8 weeks of MOTS-c treatment are enriched in processes related to fibroblast growth factors, regulation of fibrinolysis, and fibroblast growth factor receptor binding. Genes impacted by the combined intervention of aerobic exercise and MOTS-c show enrichment in cellular responses to fibroblast growth factor stimulation, fibroblast proliferation, heart morphogenesis, and collagen binding.

THBS1 emerged as a key gene modulated by all three interventions, showing enrichment in fibrosis-related pathways. RT-qPCR and WB analyses verified increased THBS1 mRNA and protein expression in diabetic rodents, which was mitigated after the interventions. THBS1 levels showed a positive association with LVPWd and LVIDs but an inverse relationship with EF, FS, and mitral inflow E/A ratio. Elevated blood glucose and TC levels in diabetic rats may induce cardiac structural changes by upregulating THBS1, thereby promoting myocardial fibrosis and impairing cardiac function. TGF-β, a downstream mediator of THBS1, directly contributes to fibrosis(Qin et al., 2015; Link et al., 2025), as evidenced by our Western blot analysis showing expression patterns consistent with THBS1. Following aerobic exercise, MOTS-c expression was upregulated in diabetic rats, suggesting a potential involvement of the MOTS-c/THBS1 Signaling Pathway in mitigating myocardial fibrosis in diabetes, in line with previous research (12, 13, 17).

In the diabetic heart, THBS1 expression is significantly elevated, contributing to interstitial fibrosis. Recent studies indicate that angiotensin II (Ang II) stimulates cardiac growth by promoting fibroblast proliferation and exacerbating cardiac fibrosis via receptor-mediated pathways such as p38 MAPK (27). The type I repeats of THBS1 bind to the latency-associated peptide (LAP) domain of TGF-β1 (28), facilitating its activation and initiating a pro-hypertrophic feedback loop that exacerbates cardiac remodeling. THBS1 has been shown to increase TGF-β1 expression and inhibit collagenase activity by activating p38 MAPK phosphorylation, leading to excessive collagen deposition in the extracellular matrix and significantly impacting myocardial function in diabetes (29). Our experimental results corroborate these findings, illustrating the activation of THBS1 and TGF-β in diabetes. Furthermore, both aerobic exercise and MOTS-c have been shown to mitigate THBS1 and TGF-β expression, thereby improving myocardial fibrosis in diabetic conditions.

**Figure 7:**
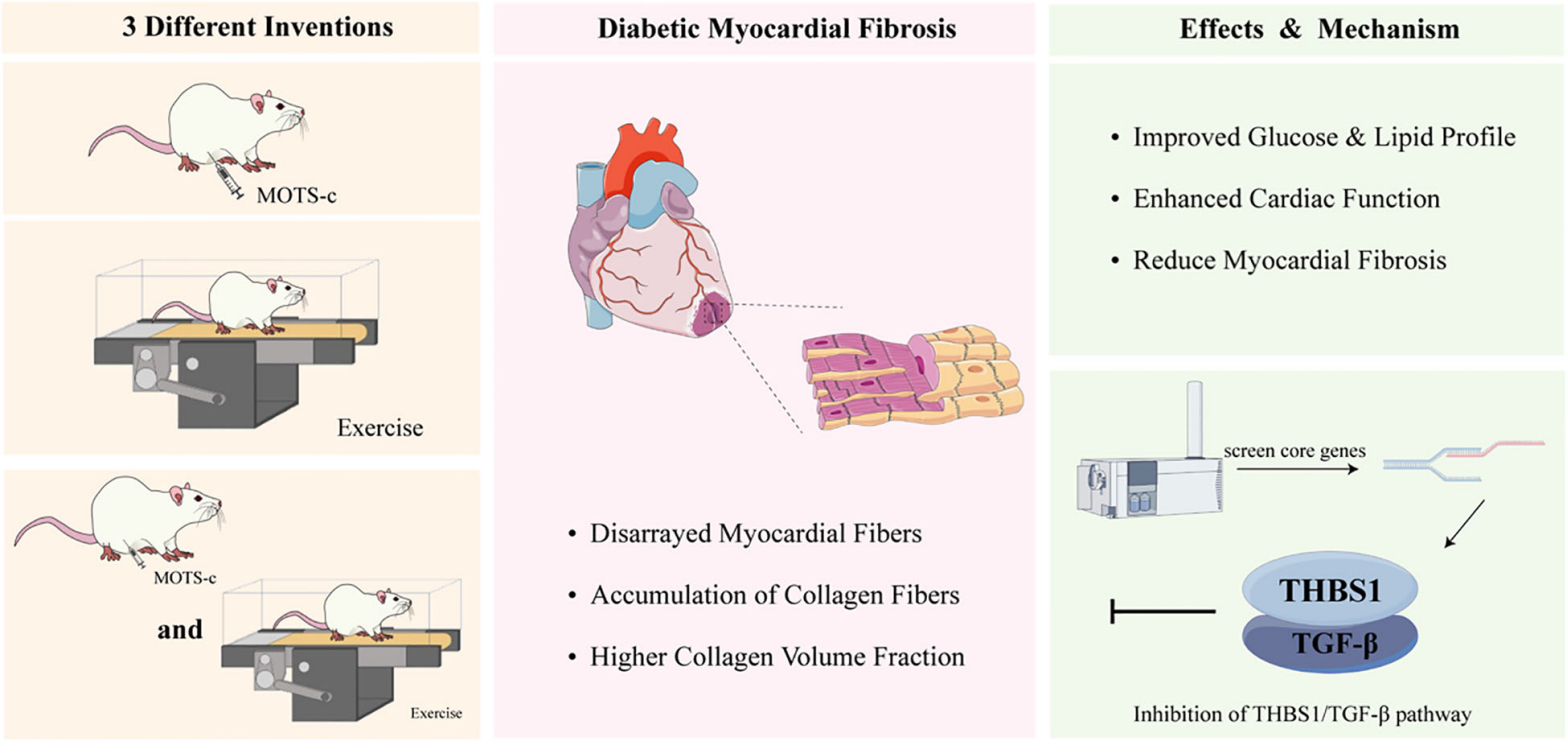
THBS1/TGF-β signaling pathway modulation by MOTS-c and exercise ameliorates diabetic myocardial fibrosis. After three different interventions, myocardial fibrosis in diabetic rats was im-proved. Through transcriptome technology, we find the THBS1/TGF-β signaling pathway was the key mechanism.

Several studies have confirmed the effectiveness of suppressing TGF-β expression and its downstream Smad proteins in alleviating fibrotic conditions (30–32). Despite TGF-β/Smad signaling being a well-established target for fibrosis treatment (30, 31), Carlos et al. observed that the absence of THBS1 didn’t significantly affect TGF-β/Smad in diabetic cardiomyopathy models (33). This suggests that THBS1’s pro-fibrotic effects in the diabetic heart may be mediated through the regulation of protease activity, potentially independent of the conventional TGF-β/Smad pathway. Further research should focus on investigating the Smad signaling cascade to clarify the specific role of MOTS-c in regulating myocardial fibrosis in diabetic patients (**Figure 7**).

In our study, myocardial fibrosis was confirmed using Masson staining and protein analysis, but we did not validate myocardial collagen expression or matrix metalloproteinase activity. Moreover, the study lacked direct validation of THBS1 as an upstream key factor through THBS1 agonists and inhibitors. Another limitation of our study is the failure to measure MOTS-c levels in serum or plasma. Future investigations would be well-advised to incorporate dynamic monitoring of circulating MOTS-c levels to paint a more comprehensive picture of the entire signaling pathway from its secretion to target organ action, thereby solidifying its reputation as an “Exercise mimetics”. While the evidence amassed in this investigation strongly underscores the pivotal role of the THBS1/TGF-β axis, we concede that the precise alterations in downstream Smad signaling remain to be directly confirmed. Future studies will concentrate on examining molecules such as p-Smad2/3, and potentially employing THBS1 agonists or antagonists, to more accurately delineate the causal contribution of each component within this pathway.

## 5 Conclusions

8 weeks of treatment with MOTS-c, regular aerobic exercise, and their combination proved to be game-changers for diabetic rats, significantly boosting their glycolipid metabolism, cutting down on collagen buildup, and giving both systolic and diastolic cardiac functions a serious shot in the arm. When they dug deeper into the transcriptomic data, researchers found that genes playing a role in myocardial fibrosis pathways were really standing out from the crowd. It appears both aerobic exercise and MOTS-c are hitting the nail on the head when it comes to easing diabetic myocardial fibrosis, all by tweaking the THBS1/TGF-β Signaling Pathway.

## Data Availability

The original contributions presented in the study are included in the article/**Supplementary Material**, further inquiries can be directed to the corresponding author/s.
