# Supplementary material for: Aerobic exercise and MOTS-c attenuate diabetic myocardial fibrosis via inhibition of the THBS1/TGF-β signaling pathway
Source: Front Endocrinol (Lausanne). 2026 Feb 3;17:1732329. doi: 10.3389/fendo.2026.1732329 (PMC12909204; doi:10.3389/fendo.2026.1732329)
Supplement: Supplementary file 2 [file Table2.docx]

**Table 1.** Baseline characteristics of rats before experiment

| **Group** | **Body Weight (g)** | **FBG (mmol/L)** | **FINS (mU/L)** | **HOMA-IR** |
| --- | --- | --- | --- | --- |
| C | 378.29±15.65 | 6.82±0.65 | 14.52±2.25 | 2.31±0.03 |
| D | 375.89±19.62 | 8.25±0.45^**^ | 10.23±1.82^**^ | 3.87±0.04^**^ |
| DE | 372.46±13.75 | 8.23±0.32^**^ | 10.43±2.01^**^ | 3.84±0.02^**^ |
| DM | 376.83±15.36 | 8.30±0.22^**^ | 10.32±1.97^**^ | 3.81±0.03^**^ |
| DME | 373.43±10.62 | 8.29±0.26^**^ | 10.29±1.86^**^ | 3.89±0.03^**^ |

^*^*p* < 0.05, ^**^*p* < 0.01, compared with group C.
